# Data science applied to the assessment of biological variation estimates

**DOI:** 10.1515/almed-2025-0042

**Published:** 2025-04-01

**Authors:** Fernando Marques-Garcia, Ana Nieto-Librero, Nerea Gonzalez-García, Xavier Tejedor-Ganduxé, Cristina Martinez-Bravo

**Affiliations:** Laboratory Medicine Department, Laboratori Clínic Metropolitana Nord, Germans Trias i Pujol University Hospital, Barcelona, Spain; Department of Statistics, Faculty of Medicine, University of Salamanca, Salamanca, Spain

**Keywords:** data science, estimates, biological variation

## Abstract

**Introduction:**

Data science is an umbrella term encompassing a set of tools and processes that make it possible to extract new information from structured or unstructured databases. This scientific discipline is gaining relevance in healthcare. In the clinical laboratory, the multiple applications of data science include the development of algorithms for obtaining population-based reference intervals or biological variation (BV) estimates. These algorithms contribute to overcoming the drawbacks of traditional or direct methods.

**Content:**

A review was performed of the state-of-the-art in algorithm-based methods for obtaining BV estimates using Real-World Data (RWD) in the field of data science.

**Summary:**

A description is provided of the structure of the algorithms currently available for obtaining BV estimates based on the scientific evidence available. An overview is provided of the advantages and drawbacks of direct methods.

**Outlook:**

The use of RWD to obtain BV estimates is a novel discipline with a considerable potential for improving our understanding of BV.

## Introduction

The concept of Big Data refers to the use of large datasets that cannot be analyzed through conventional statistical analysis processes [1]. In 2024, Big Data was defined in the Medical Subject Headings (MeSH) as: “*Extremely large amounts of data which require rapid and often complex computational analyses to reveal patterns, trends, and associations, relating to various facets of human and non-human entities*” (https://www.nlm.nih.gov/mesh/meshhome.html). The features of Big Data are referred to as the V’s [2], of which three are widely used: volume, velocity, and variety [3]. The population-based databases generated in clinical laboratories contain large datasets that contribute to reducing uncertainty and increasing precision in the statistical models used. These databases are built rapidly owing to the vast volume of data generated and gathered on daily routine. They are also characterized by their heterogeneity, which makes it possible to study both the general population and minority groups, provided that a sufficient amount of data is available for the latter.

The set of tools used for the analysis of Big Data is called “data mining”. MeSH (2024) defines it as*:* “*Use of sophisticated analysis tools to sort through, organize, examine, and combine large sets of information*” (https://www.nlm.nih.gov/mesh/meshhome.html).

The concept of Real-World Data (RWD) was recently coined to refer to this type of analysis [4], 5]. The term RWD is defined by the Food and Drugs Administration (FDA) as: *“data related to patient health status and/or the delivery of health care routinely collected from a variety of sources. Examples of RWD include data from electronic health records, claims, patient registries and data collected from other sources (such as digital healthcare technologies) that may provide an insight into a patient health status”* (https://www.nlm.nih.gov/mesh/meshhome.html). These terms and concepts are encompassed under the umbrella of Data Science, which has progressively gained relevance in clinical practice and which use is estimated to increase in the coming years [6]. Data Science is defined by MeSH as: *“An interdisciplinary field involving processes, theories, concepts, tools, and technologies, that enable the review, analysis, and extraction of valuable knowledge and information from structured and unstructured (raw) data” (* https://www.nlm.nih.gov/mesh/meshhome.html).

Laboratory information systems (LIS) store vast amounts of data initially collected for the diagnosis, monitoring or follow-up of different diseases. These datasets include laboratory results reported by clinical laboratories and other information such as demographic (sex, age) and clinical data, among other. The data initially collected to answer a clinical question can be reused to obtain hidden information that will respond to questions raised in the field of Data Science. One of the strengths of LIS is that information is stored in a (totally or partially) structured format, thereby enabling the scientific community to access and exploit data.

Classic clinical trials have traditionally adopted a direct or prospective approach. In the clinical laboratory, population-based reference intervals (pRI) [7] and biological variation (BV) estimates [8] were developed using these methods. The advent of Data Science has brought about a change in perspective, allowing for the conduction of indirect or retrospective studies. New strategies based on the use of large datasets have been developed to obtain pRI [9], 10], BV estimates [11] and for patient-based real-time quality control [12], among others.

This review is focused on obtaining BV estimates from LIS-stored databases and using data science tools. This is a novel approach for obtaining BV estimates [13].

## Biological variation estimates

At steady state condition, measurand concentrations undergo both, predictable variations such as those related to circadian rhythms, and measurable random variations such as biological variation (BV) [14].

BV is defined as the fluctuation of measurands around its homeostastic set point [15]. BV consists of two components: Within-subject BV (CV_I_) and between-subject BV (CV_G_).

Within-subject BV (CV_I_) describes variation in the concentration/activity of a measurand around a homeostatic set point within an individual [13]. In contrast, between-subject BV (CV_G_) denotes variation between the homeostatic set points of different individuals of the population [13].

The values of these estimates vary according to the homeostasis of each analyte [16], added to other factors including age, sex, or the physiopathological condition of the individual [17].

The first BV database was developed by Ricos et al. [18]. This database included estimates for 316 measurands obtained from different bibliographic sources (historical database-SEQC^ML^). In 2019, a new BV database (current database) was developed by the Task Group on Biological Variation Database (TG-BVD) of the European Federation of Clinical Chemistry and Laboratory Medicine (EFLM), in collaboration with the Working Group on Biological Variation (WG-BV) [19]. In this database, the quality of the publications was appraised according to the Biological Variation Data Critical Appraisal Checklist (BIVAC). The global estimate was derived from a meta-analysis of publications categorized using the BIVAC criteria (grade A, B, C or D) [20].

BV estimates have multiple applications in the clinical laboratory, including the development of analytical performance specifications (APS) [21] and the estimation of individual homeostatic set points (HSP) [16]; reference change values (RCV) [13]; indices of individuality (II) [22]; and personalized reference intervals (perRI) [23].

## Characteristics of direct methods

BV estimates are most commonly calculated using direct methods, with the Fraser and Harris method being the most widely used [24]. These methods are characterized by a strict control of the individuals included in the study (“normal individuals”). To such purpose, individuals are subjected to health surveys and laboratory testing [25]. “Normal” population has always been defined as healthy population. However, the concept of health “is a relative condition lacking a universal definition” and a standardized definition has not yet been established for this term [26]. Manrai et al. [26] reported the results of the US Centers for Disease Control and Prevention’s National Health and Nutrition Examination Survey (NHANES) 2013–2014 survey. This Survey provides three definitions of health: absence of common disease conditions; individuals aged 18–40 years; and overall excellent self-rating of health. Only 5 % of respondents met all the three criteria. This finding illustrates the difficulty in selecting an apparently normal population. In addition, the Fraser and Harris method is based on three major assumptions: BV data follow a normal distribution; variances are homoscedastic; and the absence of data trends (population at a steady state). When using this method, samples must be run in duplicate using the nested analysis of variance (ANOVA) method to obtain the estimates [20].

## Publications providing biological variation estimates obtained by indirect methods

It has not been until a few years ago that indirect studies, or RWD approaches, have started to be used to obtain BV estimates. To date, only five publications are available on the use of RWD for obtaining BV estimates, which indicates a limited level of scientific evidence. The publications available to date include: Loh et al., who calculated BV estimates in pediatric patients [27]; Jones, who obtained estimates for different biochemical and hormone (sex and adrenal) parameters in adult individuals [28], 29]; and Marqués-Garcia et al., who developed a novel RWD-based model to calculate BV estimates to enhance the robustness of the methods currently available [11]. The four publications only provide CV_I_ estimates for the measurands included. Loh et al. used a RWD-based method for obtaining CV_G_ estimates [30].

## Characteristics of RWD studies providing biological variation estimates

Indirect RWD-based methods reuse diagnostic and follow-up data to extract new insights, such as BV estimates in our case [9]. These methods emerge as an alternative to direct methods such as that of Fraser and Harris [24]. The definition of the database to be exploited is the first aspect to be considered before the statistic analysis is performed. The first step is to select the population to be included in the databases, being primary care patients the most stable population for this type of studies. Depending on the type of analysis to be performed, a database either of inpatients or/and ambulatory patients will be required. Such is the case of BV estimates for groups of carriers of a specific disease. The application of direct methods to unhealthy populations is more challenging owing to their frailty. It is necessary that BV estimates are available for the unhealthy population to avoid using BV estimates obtained from healthy individuals [31]. The use of a heterogeneous database will make it possible to obtain solid evidence in RWD-based studies. The inclusion of more than one hospital will provide an overview of the effect of geographical distribution on individuals (as in the study by Marqués-García et al., which included three hospitals [11]) or the analytical platform used. Long-term studies (minimum 12–18 months) facilitate access to a larger number of sources of variability in the study [11]. The number of individuals included in the analysis is not an issue, given the large size of the databases used. If RWD-based models are taken as a reference for obtaining pRI, it is necessary to include at least 10,000 individuals from the total population [10], and 400 individuals for each subgroup of the total population [32]. The possibility of categorizing the database into subgroups is a very relevant strength of RWD-based methods. Hence, the total population can be categorized by age, sex or other criteria. In contrast, this type of classification cannot be performed when direct methods are used, as they involve small samples of the population. As these studies collect long-term results, it is essential to ensure the stability of results through quality assurance. This is achieved through the evaluation of the results of internal and external quality control programs.

It is also necessary to clean the database from outliers to prevent interference with BV estimates. Although inclusion and exclusion criteria are used throughout the database generation process, we cannot ascertain that all individuals are healthy [9]. For this reason, potentially pathological values should be identified and eliminated to ensure that only data from healthy individuals are included. Different methods are available for the elimination of outliers, such as the Tukey method [27], 30], or more biological methods, such as the reference change value (RCV) [11]. Loh et al. applied the Tukey method to eliminate outliers [27], 30]. Marqués-García et al. compared the outlier elimination method based on the use of interquartile ranges against the RCV method. The authors concluded that RCV is more effective in eliminating outliers, as it includes a biological component in the formula [11]. An alternative is not eliminating outliers and using statistical methods to separate data of healthy individuals from those of unhealthy population. An example is the Bhattacharya [33] method adopted by Jones et al. in their two published studies [28], 29].

Finally, once database filtration has been performed, statistical methods are used to obtain BV estimates. Statistical methods can be classified into parametric and non-parametric methods. Parametric methods are used to describe the central distribution of data. Non parametric methods include non-linear regression models based on smoothing cubic splines and non-parametric bootstrap methods. Jones et al. used the parametric approach to obtain the CV_T_ value by the Bhattacharya method. The authors calculated the CV_I_ estimate as the difference between the CV_T_ value and the analytical coefficient of variation (CV_A_). In contrast, Loh et al. [27], 30] and Marqués-García et al. [11] used non-parametric methods. The two methods calculated the individual CV_I_ value as the difference between the CV_T_ value and the CV_A_ value. The difference between the two lies on the strategy used to obtain the global CV_I_ estimate of the population. Loh et al. used smoothing cubic splines to obtain the global CV_I_ as the median of the individual CV_I_. In turn, Marqués-García et al. used the non-parametric bootstrap method, which involves resampling datasets of a similar size by calculating the median CV_I_ in these groups.

Which of the three existing algorithms is the most suitable is still a matter of debate. Interestingly, Marqués-García et al. [11] corrected many of the limitations reported in previous studies. For example: the authors conducted a multicentric study that included a higher volume of data. Outliers were eliminated using a more biological and less statistical approach (as the RCV). As a result, the estimates obtained showed a good correlation with the EFLM biological variation database [19]. Finally, the global CV_I_ was obtained by the bootstrap method, which enabled them to calculate the confidence intervals for the estimates. Loh et al. published the only study to use a RWD-based method to obtain CV_G_ estimates [30]. The authors adapted the Fraser and Harris’ formula [24] to large datasets to estimate the CV_G_.

## Advantages and drawbacks of RWD-based methods for obtaining biological variation estimates

The use of algorithms based on database analysis to obtain BV estimates represents a different approach to that of direct methods and overcomes the limitations of the latter. In RWD-based methods, variability in the database is assessed using laboratory results obtained under real-life working conditions rather than under ideal conditions, as in direct methods. Additionally, RWD methods are less invasive, as they involve reusing data; therefore, regular blood draws are not required as in direct methods. As compared to RWD methods, direct methods involve higher costs, as they require specific sample extraction material, reagents, and facilities, among others. Another aspect to be considered in direct studies is the concept of normality. As direct methods are based on a small sample of the population, the presence of subclinical conditions may influence BV estimates. In contrast, when large databases are analyzed by RWD methods, this effect is minimized. The values of the estimates obtained by RWD methods show a good correlation with those obtained using direct methods [11]. This finding demonstrates the power of these algorithms to separate signal (BV) from noise.

RWD strategies have some drawbacks that will have to be overcome in the future. To date, electronic health record data has not yet been standardized either at national or international level [34], although some efforts are being made. The use of standardized electronic health records (EHRs) in all territories would enable the homogeneous exploitation of data. Hence, EHR standardization would make it possible to obtain databases with a similar structure and use an international nomenclature such as LOINC or SNOMED-CT. Additionally, a higher level of standardization will enhance the quality of the data available. Another limitation is data protection. Efforts should be made to enhance data anonymization to safeguard the confidentiality of patient information [35]. An effective approach would be to build secure inter-hospital networks that provide anonymized data and enable a secure circulation of data. Statistical analysis involves complex procedures that require advanced skills and access to specific software tools. The level of evidence currently available for the use of RWD-based methods to obtain BV estimates is low, as only five studies have been published to date. Efforts should be intensified to develop algorithms that yield robust BV estimates. The studies currently available do not report data stability (data at steady state), a matter that should be addressed in future studies. The data collection period should be extended to include as many sources of variability as it is possible.

## Conclusions

Interest is growing in the application of Real-World Data (RWD) to healthcare. RWD-based methods have been used in the recent years to obtain population-based reference intervals. Other novel applications of RWD include patient-based real-time quality control, and the calculation of BV estimates. The RWD approach for obtaining BV estimates overcomes the drawbacks of direct methods. Specifically, RWD methods have a lower cost and are less time-consuming, although data management and statistical analysis are more complex, as compared to direct methods. Although a multicenter study is available in the literature, extending the number of centers included in these studies would make it possible to identify differences in BV estimates across geographic regions or analytical platforms. Large datasets contain a vast amount of information that facilitates the segregation of the population into different subgroups. As a result, specific BV estimates can be obtained for different population subgroups. This way, estimates can be obtained for vulnerable groups such as the pediatric, elderly or unhealthy population. Although BV estimates obtained by RWD-based methods are already available, further studies are necessary to develop working algorithms that provide robust BV estimates. Access to robust BV estimates will contribute to their precise application and provide cumulative scientific evidence that will help optimize operating algorithms. The results obtained require validation to appraise their suitability. Thus, they should be compared against the current gold standard, the EFLM BV database [19]. In Spain, the ongoing Spanish Multicentric Project (BiVaBiDa) is focused on the development of RWD-based applications for obtaining BV estimates for different groups of interest.
